# The impact of ethnicity on stroke care access and patient outcomes: a New Zealand nationwide observational study

**DOI:** 10.1016/j.lanwpc.2021.100358

**Published:** 2022-01-03

**Authors:** Stephanie G. Thompson, P. Alan Barber, John H. Gommans, Dominique A. Cadilhac, Alan Davis, John N. Fink, Matire Harwood, William Levack, Harry McNaughton, Valery L. Feigin, Virginia Abernethy, Jackie Girvan, Hayley Denison, Marine Corbin, Andrew Wilson, Jeroen Douwes, Annemarei Ranta

**Affiliations:** a Department of Medicine, University of Otago, PO Box 7343, Wellington 6242, New Zealand; b University of Auckland, Private Bag 92019, Auckland 1142, New Zealand; c Hawke's Bay District Health Board, Private Bag 9014, Hastings 4156, New Zealand; d Monash University, Wellington Road, Clayton, Victoria 3800, Australia; e Whangarei Hospital, Maunu Road, Private Bag 9742, Whangarei 0148, New Zealand; f Canterbury District Health Board, PO Box 1600, Christchurch 8140, New Zealand; g University of Auckland, Private Bag 92019, Auckland 1142, New Zealand; h University of Otago Wellington, PO Box 7343, Wellington 6242, New Zealand; i Medical Research Institute of New Zealand, Private Bay 7902, Wellington 6242, New Zealand; j Auckland University of Technology, Private Bag 92006, Auckland 1142, New Zealand; k Stroke Foundation New Zealand, PO Box 12482, Wellington 6144, New Zealand; l 18 River Road, RD 7, Ashburton 7777, New Zealand; m Research Centre for Hauora and Heath, Massey University, PO Box 756, Wellington 6140, New Zealand; n Wairau Hospital, PO Box 46, Hospital Road, Blenheim 7240; o Department of Medicine, University of Otago Wellington, PO Box 7343, Wellington 6242, New Zealand

**Keywords:** Stroke, Disparities, Ethnicity, Indigenous, Health services research, Epidemiology, Outcome resarch

## Abstract

**Background:**

Ethnic inequities in stroke care access have been reported internationally but the impact on outcomes remains unclear. In New Zealand, data on ethnic stroke inequities and resultant effects on outcomes are generally limited and conflicting.

**Methods:**

In a prospective, nationwide, multi-centre observational study, we recruited consecutive adult patients with confirmed stroke from 28 hospitals between 1 May and 31 October 2018. Patient outcomes: favourable functional outcomes (modified Rankin Scale 0-2); quality of life (EQ-5D-3L); stroke/vascular events; and death at three, six and 12 months. Process measures: access to reperfusion therapies, stroke-units, investigations, secondary prevention, rehabilitation. Multivariate regression analyses assessed associations between ethnicity and outcomes and process measures.

**Findings:**

The cohort comprised 2,379 patients (median age 78 (IQR 66-85); 51·2% male; 76·7% European, 11·5% Māori, 4·8% Pacific peoples, 4·8% Asian). Non-Europeans were younger, had more risk factors, had reduced access to acute stroke units (aOR=0·78, 95%CI, 0·60-0·97), and were less likely to receive a swallow screen within 24 hours of arrival (aOR=0·72, 0·53-0·99) or MRI imaging (OR=0·66, 0·52-0·85). Māori were less frequently prescribed anticoagulants (OR=0·68, 0·47-0·98). Pacific peoples received greater risk factor counselling. Fewer non-Europeans had a favourable mRS score at three (aOR=0·67, 0·47-0·96), six (aOR=0·63, 0·40-0·98) and 12 months (aOR=0·56, 0·36-0·88), and more Māori had died by 12 months (aOR=1·76, 1·07-2·89).

**Interpretation:**

Non-Europeans, especially Māori, had poorer access to key stroke interventions and experience poorer outcomes. Further optimisation of stroke care targeting high-priority populations are needed to achieve equity.

**Funding:**

New Zealand Health Research Council (HRC17/037).

**Research in context**

**Evidence before this study**

Prior research has shown that ethnic minority groups have poorer access to a range of best practice stroke interventions. The effect of ethnicity on outcomes post stroke is less clear. In New Zealand there is limited and conflicting evidence on ethnic inequities for patients with stroke.

**Added value of this study**

This large, nation-wide, prospective observational study using high-quality, consecutive patient-level data shows poorer outcomes for non-Europeans post stroke and demonstrates evidence for unequal access to some key acute and rehabilitation stroke care interventions.

**Implications of all the available evidence**

This study along with previous international research confirms that inequities in access to best practice stroke care by ethnicity exist. These ethnic disparities in access can explain, at least in part, the poorer outcomes observed, and suggests that further optimisation of stroke care, targeting high-priority populations, is needed to achieve equity and equality of outcomes.

Alt-text: Unlabelled box

## Introduction

Stroke is a leading cause of death and disability.^1^ International research has provided evidence that ethnic minorities often have poorer access to best practice stroke care.^2^^,^^3^ Research on patient outcomes in different ethnic groups has produced conflicting results.^2^^,^^4^, ^5^, ^6^ New Zealand is a multi-ethnic society with 70·2% identifying as European, 16·5% indigenous Māori, 15·1% Asian and 8·1% with Pacific Island backgrounds. Many identify with more than one ethnic group and 27·4% were not born in New Zealand.^7^ This multi-ethnic population facilitates assessment of ethnic differences in access to healthcare and the associations with outcomes. Māori and Pacific peoples experience stroke at a younger age and have a rate of decline in stroke incidence and one-year mortality that is slower than New Zealand Europeans.^8^ The differences in incidence have been largely attributed to a greater incidence of modifiable stroke risk factors, including hypertension, diabetes, and smoking in non-Europeans.^8^^,^^9^

While New Zealand research has provided evidence of ethnic inequities in general health care access and patient outcomes, there is little information on stroke care access by ethnicity, and studies assessing the effect of ethnicity on outcome post stroke have shown conflicting results.^8^, ^9^, ^10^, ^11^ Such information is necessary to better understand the role health systems and stroke services play in stroke inequities and inform the development of programmes that achieve greater equity.

## Methods

REGIONS Care (Reducing Ethnic and Geographic Inequities to Optimise New Zealand Stroke Care) is a multi-part observational study designed to assess the impact of geography and ethnicity on stroke care access and outcomes. It involves nationwide, prospectively collected, patient data with a sub-set of patients recruited to undergo extended follow up, linkage with health administrative data, focus groups and surveys. The full study methods have been described elsewhere,^12^ and are outlined briefly below. Here, we report the results of analyses focusing on ethnicity.

### Study sample

This study involved all 28 New Zealand hospitals caring for patients with acute stroke and associated rehabilitation and community services. All adult patients admitted to hospital with a discharge diagnosis of stroke between 1 May and 31 July 2018 were captured. After this date, consecutive patient recruitment continued until hospitals achieved a minimum sample size of 150 (thrombectomy centres), 100 (all other centres) or until 31 October 2018, whichever occurred first. We grouped patients by self-identified ethnicity, based on Statistics New Zealand coding:^13^ New Zealand European, Māori, Pacific peoples, Asian and Other.

Patients with transient ischaemic attack, those initially suspected to have a stroke but a final diagnosis other than stroke, including thrombolysed stroke mimics, and people aged <18 years were excluded. For each patient, only the initial admission during the study period was counted as an index event; any subsequent admissions were considered outcome events.

### Data collection

Data were primarily collected by front line clinical teams at the time the patient was admitted to hospital and during three-month follow-up encounters and entered into a central database. Additional follow-up data at six- and 12-months were collected by the central study team via telephone interviews supplemented by mailed questionnaires.

Baseline data included patient demographics, vascular risk factors, pre-morbid level of function, employment status, domiciliary information, disability at hospital admission, arrival mode, arrival time from symptom onset, and stroke characteristics. Post-admission data included in-hospital interventions and services, investigations to determine stroke aetiology, and therapies up to three months post admission, follow-up appointments up to 12 months, and outcome variables as described below. All patients were invited at three months to consent to further follow-ups at six and 12 months until a pre-set sample size target was reached.

### Outcomes

Stroke outcome measures included: modified Rankin Scale (mRS) score at three, six and 12 months dichotomised into favourable outcome (mRS=0-2) and unfavourable outcome (mRS=3-6); mRS shift analysis using ordinal regression analysis; five-dimension, three-level EuroQol health-related quality of life questionnaire (EQ-5D-3L) scores; stroke recurrence; vascular events; readmission; and death at three, six, and 12 months.

Stroke care process measures included: stroke reperfusion therapies (for all patients as well as those who arrived within the appropriate treatment window and with no documented contraindications), acute stroke unit care, timely assessment by key members of an inter-disciplinary stroke team, relevant investigations to determine stroke aetiology (CT, MRI, carotid imaging, ECG and telemetry), early mobilisation within 48 hours, swallow assessment within six and 24 hours, guideline-based deep vein thrombosis prophylaxis, early prescription of anti-thrombotic agents, prescription of best medical management tailored to an individual's stroke diagnosis and cause by time of discharge, life-style counselling, and timely access to, and therapist contact time, during inpatient and community rehabilitation.

### Data analysis

All data were analysed in Stata/IC 16·0. Patient baseline characteristics were summarised using proportions for dichotomous, means and standard deviations for continuous, and medians and interquartile ranges for non-normally distributed continuous variables. We used Pearson's chi-squared test to compare dichotomous, ANOVA for normally and Kruskal-Wallis rank test for non-normally distributed continuous baseline variables between ethnic groups. Logistic regression was used to assess associations between ethnicity and dichotomous outcomes, with European ethnicity as the reference group. Non-European ethnicity refers to all patients other than New Zealand Europeans. ‘Other’ ethnicity includes Middle Eastern, Latin American and African ethnicities.^13^ For the main analyses we excluded patients with missing data as this was less than 5% for baseline and process of care variables. Multivariable models were controlled for known confounders, including age, sex, stroke severity, stroke type, and pre-morbid level of function. In addition, we adjusted for hospital location (urban/non-urban) and baseline characteristics that differed (p<0.1) between groups. For specific service process measures, we included additional variables known to impact intervention access that are outside the control of the hospital service, such as hospital arrival time and mode when considering reperfusion therapy access. We then backward eliminated covariates if the impact on odds ratios was <0.1 and model fit was either improved or unaffected, starting with differences in baseline characteristics, followed by specific covariates added for specific models, and finishing with known confounders. We checked for interaction effects between hospital location and ethnicity and conducted additional multi-level mixed effects regression to adjust for clustering of patients in the same hospital.

### Study funding and ethics

The Health Research Council of New Zealand (HRC 17/037) funded this study, which received ethics approval from the Central Region Health and Disability Ethics Committee (17CEN164). Routine clinical patient data collection up to 3-months following discharge was classed as ‘clinical audit’ and the ethics committee waived the need for individual patients consent. We sought patient consent for extended follow-up and data linkage at the three-month follow-up assessment.

### Role of the funding source

The funder had no role in the design of the study, data collection, analysis or interpretation of the results, and the content of the manuscript was written independently of the funder. The corresponding author had full access to all the data and all authors agreed with the decision to submit for publication.

## Results

There were 2,379 eligible patients (mean age 75 (standard deviation (SD) 13·7); 51·2% male; 81·5% ischaemic and 12·3% haemorrhagic stroke) included during the study period: 1823 (76·7%) European, 273 (11·5%) Māori, 114 (4·8%) Pacific peoples, 115 (4·8%) Asian, and 54 (2·3%) ‘Other’. Baseline demographics, risk factors, and stroke characteristics differed significantly between ethnic groups (Table 1). Compared with Europeans, non-Europeans were younger at the time of stroke, were more likely to have diabetes or smoke, and a larger proportion were independent and were employed prior to the stroke. Māori and Pacific peoples also had higher rates of rheumatic heart disease. Follow-up data was available for 77.9% of the whole cohort at three months and 95.1% and 92.3% at six and 12 months respectively for the subset of patients who consented to extended follow-up (supplementary table 1). Differences in baseline characteristics for those with versus those without complete follow-up data (both European and non-European) are presented in supplementary tables 2-4. We explored the possibility of an important interaction between hospital location and ethnicity, however, found no significant interaction effects; therefore, hospital location and ethnicity were added as separate covariates to the regression models.

**Table 1:** Patient baseline characteristics.

	European	Māori	Pacific	Asian	Other	p value
**Patients** n (%)*	1823 (76·7)	273 (11·5)	114 (4·8)	115 (4·8)	54 (2·3)	
**Age** Median (IQR)	80 (71-87)	65 (56-75)	66 (55-76)	69 (60-80)	74·5 (64-83)	<0·0001
**Sex** n (%) Male Female	945 (51·8) 878 (48·2)	121 (44·3) 152 (55·7)	59 (51·8) 55 (48·2)	59 (51·3) 56 (48·7)	35 (64·8) 19 (35·2)	0·05
**Primary diagnosis** n (%) Ischaemic stroke Haemorrhagic stroke Stroke not specified Other	1494 (82·0) 210 (11·5) 59 (4·9) 28 (1·5)	233 (85·4) 22 (8·1) 17 (6·2) 1 (0·4)	94 (82·5) 17 (14·9) 2 (1·8) 1 (0·9)	78 (67·8) 34 (29·6) 3 (2·6) 0 (0·0)	38 (70·4) 9 (16·7) 5 (9·3) 2 (3·7)	<0·0001
**Ischaemic stroke location** n (%) † Anterior circulation Posterior circulation Spinal cord Other Unknown	978 (68·6) 348 (24·4) 3 (0·2) 21 (1·5) 76 (5·3)	168 (74·3) 49 (21·7) 1 (0·4) 4 (1·8) 4 (1·8)	59 (63·4) 31 (33·3) 0 (0·0) 1 (1·1) 2 (2·2)	49 (66·2) 21 (28·4) 0 (0·0) 2 (2·7) 2 (2·7)	22 (61·1) 10 (27·8) 1 (2·8) 1 (2·8) 2 (5·6)	0·09
**Ischaemic stroke cause** n (%) † Cardioembolic – AF Carotid stenosis Vertebrobasilar stenosis Small vessel Intracranial stenosis Other Unknown Cardioembolic – non-AF Dissection	490 (32·8) 78 (5·3) 20 (1·4) 208 (14·0) 20 (1·4) 52 (3·5) 590 (39·7) 21 (1·4) 7 (0·5)	83 (35·6) 6 (2·5) 1 (0·4) 32 (13·5) 8 (3·4) 13 (5·5) 87 (36·7) 7 (3·0) 0 (0·0)	27 (28·7) 5 (5·4) 1 (1·1) 19 (20·4) 2 (2·2) 5 (5·4) 32 (34·4) 2 (2·2) 0 (0·0)	20 (25·6) 5 (6·4) 3 (3·9) 14 (18·0) 3 (3·9) 6 (7·7) 26 (33·3) 0 (0·0) 1 (1·3)	7 (17·1) 2 (4·9) 0 (0·0) 4 (9·8) 0 (0·0) 4 (9·8) 23 (56·1) 0 (0·0) 1 (2·4)	0·02
**Haemorrhagic stroke location** n (%) ‡ Lobar Deep Other Unknown	106 (53·5) 71 (35·9) 17 (8·6) 4 (2·0)	11 (52·4) 7 (33·3) 3 (14·3) 0 (0·0)	6 (37·5) 8 (50·0) 1 (6·3) 1 (6·3)	8 (24·2) 24 (72·7) 1 (3·0) 0 (0·0)	5 (55·6) 4 (44·4) 0 (0·0) 0 (0·0)	0·05
**Haemorrhagic stroke cause** n (%)‡ Hypertensive Anticoagulation Haemorrhagic transformation Trauma Other Unknown Amyloid Angiopathy Underlying SOL/AVM/aneurysm	99 (50·8) 12 (6·2) 23 (11·8) 1 (0·5) 4 (2·1) 36 (18·5) 15 (7·7) 5 (2·6)	9 (40·9) 0 (0·0) 7 (31·8) 0 (0·0) 2 (9·1) 3 (13·6) 0 (0·0) 1 (4·6)	11 (68·8) 1 (6·3) 2 (12·5) 0 (0·0) 1 (6·3) 1 (6·3) 0 (0·0) 0 (0·0)	24 (70·6) 0 (0·0) 2 (5·9) 0 (0·0) 2 (5·9) 6 (17·7) 0 (0·0) 0 (0·0)	28 (65·1) 0 (0·0) 4 (9·3) 0 (0·0) 2 (4·7) 9 (20·9) 0 (0·0) 0 (0·0)	0·29
**Arrival mode** n (%) Hospital transfer Ambulance – road Ambulance – helicopter Ambulance (non-emergency) Private vehicle Not documented	63 (3·6) 848 (48·2) 28 (1·6) 366 (20·8) 320 (18·2) 136 (7·7)	8 (3·0) 146 (54·5) 9 (3·4) 26 (9·7) 65 (24·3) 14 (5·2)	7 (6·3) 59 (52·7) 0 (0·0) 9 (8·0) 30 (26·8) 7 (6·3)	5 (4·4) 56 (49·1) 0 (0·0) 12 (10·5) 30 (26·3) 11 (9·7)	1 (1·9) 19 (36·5) 1 (1·9) 12 (25·0) 12 (23·1) 6 (11·5)	<0·0001
**Delay in arrival** n (%) <4 hours <24 hours	780 (43·8) 1380 (77·8)	120 (44·9) 200 (75·2)	47 (41·6) 81 (71·7)	49 (42·6) 84 (73·0)	24 (45·3) 39 (73·6)	0·98 0·38
**Initial observations** Median (IQR) Blood glucose Systolic blood pressure	7·0 (5·9-8·7) 161 (140-184)	7·1 (6·1-9·9) 150 (132-175)	7·5 (6·1-10·5) 155 (140-180)	7·6 (6·2-9·6) 165 (145-185)	6·7 (5·7-9·3) 168 (141-182)	0·05 0·0001
**Risk factors** n (%) Prior vascular event Stroke TIA Hypertension Diabetes Dyslipidaemia AF Smoker Ischaemic heart disease Rheumatic heart disease Family history of stroke Carotid stenosis	391 (21·7) 250 (13·9) 1294 (71·5) 359 (19·9) 745 (41·5) 638 (35·3) 159 (8·8) 471 (26·2) 20 (1·1) 113 (6·3) 146 (8·2)	67 (25·0) 32 (11·9) 190 (70·1) 97 (35·8) 122 (45·9) 107 (40·4) 99 (37·2) 60 (22·5) 16 (6·0) 30 (11·4) 17 (16·5)	22 (19·3) 8 (7·0) 89 (78·1) 55 (48·3) 59 (51·8) 28 (24·6) 20 (18·2) 20 (17·5) 4 (3·5) 3 (2·6) 5 (4·5)	22 (19·3) 6 (5·2) 83 (73·5) 44 (38·6) 48 (42·1) 22 (19·5) 8 (7·0) 11 (9·7) 0 (0·0) 9 (7·8) 7 (6·1)	13 (24·1) 7 (13·5) 39 (72·2) 16 (29·6) 24 (44·4) 12 (22·6) 1 (1·9) 13 (24·1) 0 (0·0) 6 (11·3) 5 (9·4)	0·40 0·007 0·27 <0·0001 <0·0001 <0·0001 <0·0001 <0·0001 <0·0001 0·001 <0·0001
**Reason for no anticoagulation for AF**§ n (%) Falls ICH, GI bleed, other bleed Frailty, comorbidities, side effects Pre-/peri-procedure Patient preference/non-compliant Stopped for procedure/not restarted AF duration felt not significant Unknown	11 (6·5) 36 (21·3) 25 (14·8) 6 (3·6) 30 (17·8) 6 (3·6) 5 (3·0) 50 (29·6)	2 (10·0) 3 (15·0) 1 (5·0) 1 (5·0) 5 (25·0) 1 (5·0) 0 (0·0) 7 (35·0)	0 (0·0) 1 (16·7) 1 (16·7) 0 (0·0) 0 (0·0) 0 (0·0) 0 (0·0) 4 (66·7)	0 (0·0) 1 (25·0) 0 (0·0) 0 (0·0) 1 (25·0) 0 (0·0) 0 (0·0) 2 (50·0)	1 (16·7) 1 (16·7) 0 (0·0) 0 (0·0) 1 (16·7) 0 (0·0) 0 (0·0) 3 (50·0)	0·99
**Pre-stroke situation** Pre-stroke mRS Independent (0-2) Dependent (3-5) Employed Residence Home alone Home with others Residential care Other	1540 (85·2) 268 (14·7) 305 (16·8) 599 (32·9) 1041 (57·2) 165 (9·1) 15 (0·8)	246 (92·5) 20 (7·3) 80 (30·2) 49 (18·0) 210 (76·9) 7 (2·6) 7 (2·6)	105 (92·9) 8 (7·0) 38 (33·6) 7 (6·1) 105 (92·1) 2 (1·8) 0 (0·0)	103 (92·8) 8 (7·2) 25 (22·1) 15 (13·0) 97 (84·4) 2 (1·7) 1 (0·9)	46 (85·2) 8 (14·8) 17 (31·5) 11 (20·4) 38 (70·4) 2 (3·7) 3 (5·6)	0·001 <0·001 <0·001
**Level of disability on arrival** GCS verbal <5 Requires assistance to walk Arms MRC <3/5	638 (35·1) 1011 (55·7) 639 (35·2)	105 (38·5) 155 (56·8) 114 (41·8)	46 (40·4) 60 (52·6) 48 (42·1)	47 (40·9) 75 (65·2) 54 (47·0)	22 (40·7) 31 (57·4) 16 (29·6)	0·41 0·32 0·01

AF=atrial fibrillation; SOL=space occupying lesion; AVM=arteriovenous malformation; IQR=interquartile range; TIA=transient ischaemic attack; BMI=body mass index; ICH=intracerebral haemorrhage; GI=gastrointestinal; mRS=modified Rankin Scale; GCS=Glasgow Coma Scale; MRC=Medical Research Council.

⁎ The denominator for % in the ‘Patients’ row comprises the entire study cohort. For remaining variables, unless otherwise specified, the denominator is the total number of participants within the ethnic group described in each column.

† The denominator is the number of ischaemic strokes in each ethnic group.

‡ The denominator is the number of haemorrhagic strokes in each ethnic group.

§ The denominator comprises patients with known diagnosis of AF at time of hospital presentation.

### Impact of ethnicity on access to stroke interventions

**Figure 1:**
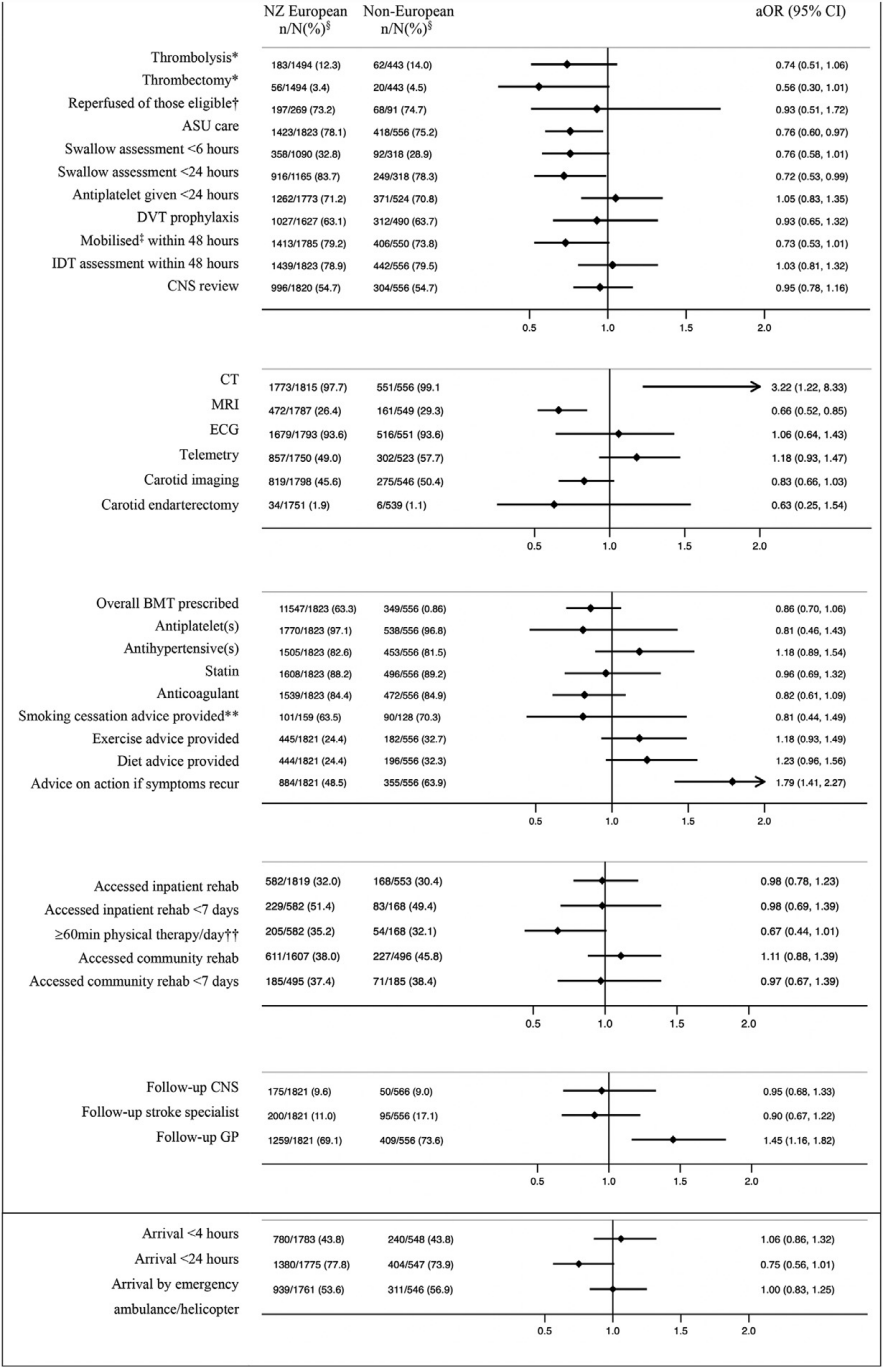
Access to stroke interventions/care by ethnicity. aOR=adjusted odds ratio (all outcomes were adjusted for pre-morbid level of function, age sex, rurality, stroke severity, baseline characteristic differences of p<0.1, and intervention specific covariates such as time delay to reach hospital, mode of transport for reperfusion therapies and palliation within 24 hours for early mobilisation and allied health input. Covariates were backward eliminated unless removal substantially impacted odds ration aiming to minimise number of covariates and optimise model fit); 95% CI=95% confidence interval; ASU=acute stroke unit; DVT=deep vein thrombosis; IDT=interdisciplinary team; BMT=best medical therapy; CNS review refers to a stroke clinical nurse specialist review of the patient on the ward while an inpatient; BMT=’best medical therapy’ refers to antiplatelet(s), statins, and anti-hypertensives for non-cardioembolic ischaemic stroke patients, anti-hypertensives for ICH patients attributed to hypertension, and anticoagulation for patients with cardioembolic stroke unless any contraindications documented; GP=general practitioner; Follow-up with stroke nurse refers to post-discharge follow-up appointment with a stroke clinical nurse specialist. § n/N(%): the numerator refers to the number of people that received the intervention and the denominator to the number of people for whom we had data available. *Denominator for these analyses consists of only those patients with a primary diagnosis of ischaemic stroke; †‘Reperfused of those eligible’ refers to patients undergoing thrombolysis and/or thrombectomy among those who presented within the require time window and did not have appropriate exclusion criteria; ‡’Mobilised’ refers to any ‘out of bed activity’; ** Analysis limited to current smokers at the time of presentation; ††weekdays only.

There was no association between ethnicity and arrival to hospital within 4 or 24 hours, or in treatment with intravenous thrombolysis (IVT) (Figure 1). The proportion of patients with ischaemic stroke treated with IVT was: Māori (16·3%), Pacific Peoples (17·0%), Europeans (12·3%), and Asians (7.7%) (Supplementary Figure 1). There was a non-significant trend among non-Europeans toward poorer access to endovascular thrombectomy (EVT) (aOR=0·56, 0·30-1·01); however, when limiting the analysis to those eligible for reperfusion therapies, we found no differences among ethnic groups.

Non-Europeans, especially Māori, were less likely to be treated in an acute stroke unit (ASU) compared to Europeans (aOR=0·68, 0·51-0·91; Supplementary Figure 1). Non-Europeans were also less likely to receive a swallow screen within 24 hours of hospital arrival (aOR=0·57, 0·33-0·99). There was no association between ethnicity and interdisciplinary assessment within 48 hours. We found non-significant associations between non-European ethnicity and likelihood of mobilisation within 48 hours (aOR=0·73, 0·53-1·01) and between Māori ethnicity and receiving appropriate deep vein thrombosis (DVT) prophylaxis (aOR=0·77, 0·56-1·05). Pacific peoples were more likely to be reviewed by a clinical nurse specialist (CNS) during their hospital admission (aOR=1·51, 1·01-2·26). Non-Europeans were more likely to access CT scanning (aOR=3·22, 1·22-8·33), Pacific peoples were less likely to have an MRI scan (aOR=0·41, 0·25-0·68), while Asians were more likely to have telemetry (aOR=1·76, 1·13-2·73). There were no associations between ethnicity and having an ECG or carotid endarterectomy; however, non-Europeans trended towards a lower likelihood of undergoing carotid imaging (aOR=0·83, 0·66-1·03). Māori with a cardioembolic source of stroke were less likely to be discharged on anticoagulant medication than Europeans (aOR=0·62, 0·41-0·94); there were no other associations between ethnicity and secondary prevention medication prescription. Pacific peoples were more likely to receive lifestyle modification counselling, including diet and exercise advice, and all non-Europeans were more likely to receive counselling on what to do if symptoms were to recur. There was no association between ethnicity and smoking cessation education.

There was no association between ethnicity and overall access to inpatient or community rehabilitation or reaching these services within seven days of acute hospital admission or discharge, respectively. There was a strong trend toward non-Europeans having poorer access to at least one hour of physical therapy per day during inpatient rehabilitation (aOR=0·67, 0·44-1·00). Unadjusted, non-parametric tests for number of post-discharge community rehabilitation team contacts found that compared with a median (inter-quartile range) of 3 (1-8) contacts in Europeans, Māori had 5 (1-13; p=0·04), Pacific 4·5 (2-11; p=0·05), and Asians 6 (2-12·5; p=0·02) contacts. Non-Europeans overall, and Pacific peoples and Asians specifically, were more likely to be followed up by their general practitioner (GP) after discharge from hospital (aOR=1·66, 1·05-2·63 and aOR=1·73, 1·11-2·71 respectively) (Figure 1).

Of the 249 Māori patients in the cohort, 153 (61·5%) were offered Māori/tikanga cultural support services during their hospital admission and 114 (45·7%) accessed these services. For Pacific patients, 31 of 101 (30·7%) were offered Pacific support services, and 17 (16·8%) accessed the service.

### Impact of ethnicity on outcomes

**Figure 2:**
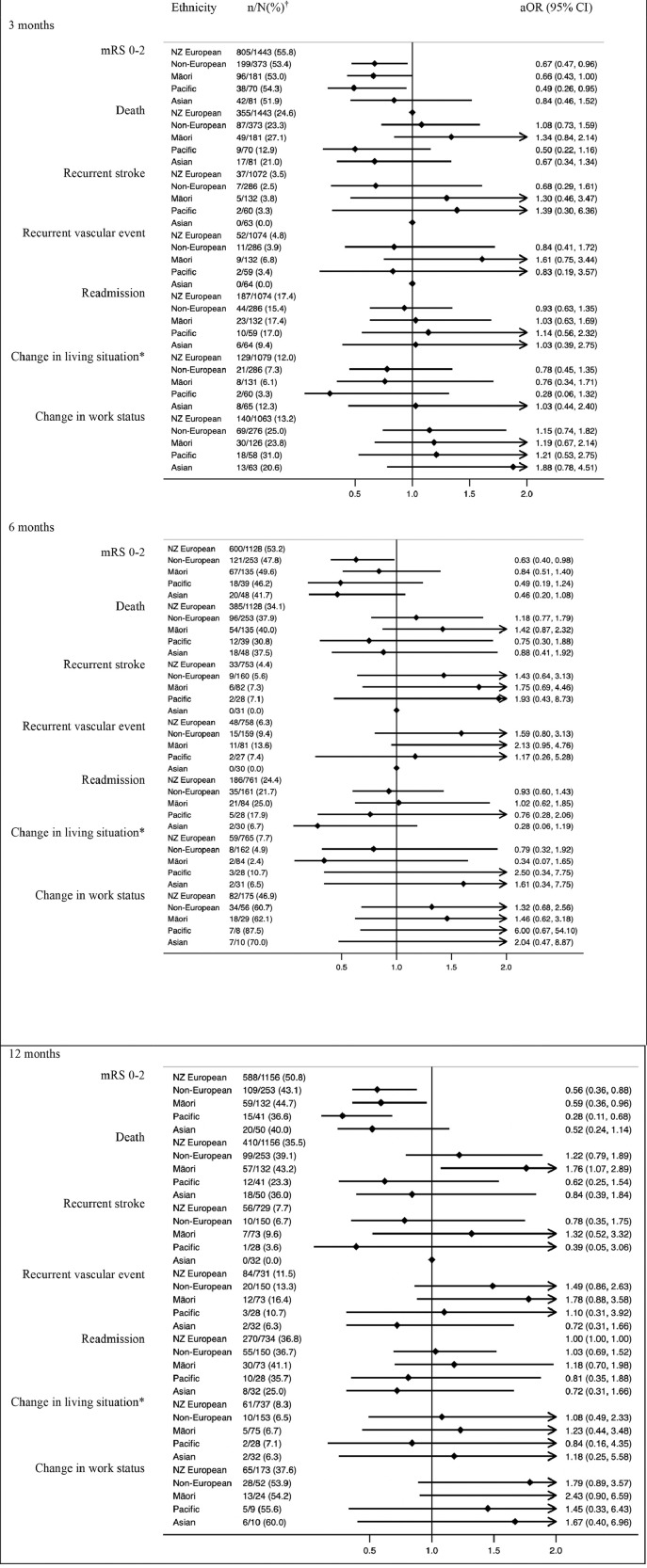
Stroke outcomes by ethnicity. 95% CI= 95% confidence interval; aOR = adjusted odds ratio (all outcomes were adjusted for pre-morbid level independence, age, ethnicity, stroke severity, and baseline characteristic differences of p<0.1. Covariates were backward eliminated if removal did not substantially impact the odds ratio aiming to minimise number of covariates and optimise model fit); mRS=modified Rankin Scale; †n/N(%): the numerator refers to the number of people who achieved an outcome and the denominator refers to the number of people for whom data was available had data available; *‘Change in living situation’ refers to a new move to a care facility, move from independent living to a family member or other carer home, or a family member or carer moving into the patient's home to provide care.

**Figure 3:**
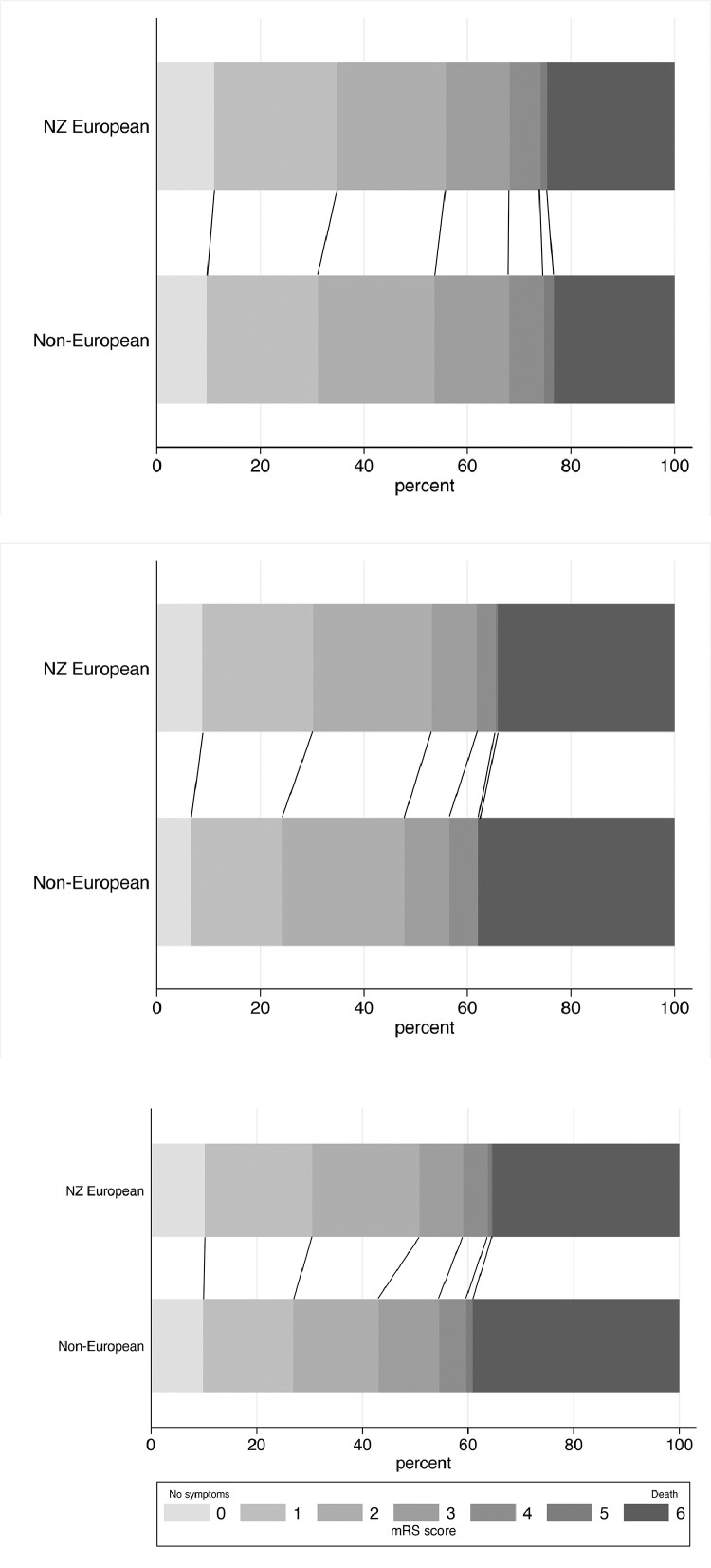
mRS shift analysis at 3, 6 and 12 months.

Non-Europeans were less likely to achieve functional independence (mRS 0-2) at three months (aOR=0·67, 0·47-0·96), six months (aOR=0·63, 0·40-0·98) or 12 months (aOR=0·56, 0·36-0·88) (Figure 2). The mRS shift analysis, showing a comparison of the distribution of mRS scores for European and non-European patients at the three time points, is shown in Figure 3. These differences were driven primarily by results for Māori and Pacific peoples who had significantly reduced odds of a favourable outcome at three months (aOR=0·66, 0·43-0·999 and aOR=0·49, 0·26-0·95 respectively) and 12 months (aOR=0·59, 0·36-0·96 and aOR=0·28, 0·11-0·68 respectively). Māori had higher odds of death at twelve months (aOR=1·76, 1·07-2·89).

No significant differences were observed in stroke or vascular event recurrence between Europeans or non-Europeans at any of the three time-points. Overall, recurrent event rates were low. Māori had non-significant trends toward higher odds of recurrent stroke and vascular events at each time point. Among Asian patients followed up until twelve months, none had a recurrent stroke, and they also had the lowest rate of recurrent vascular events (6·3%) at 12 months of any ethnic group.

**Figure 4:**
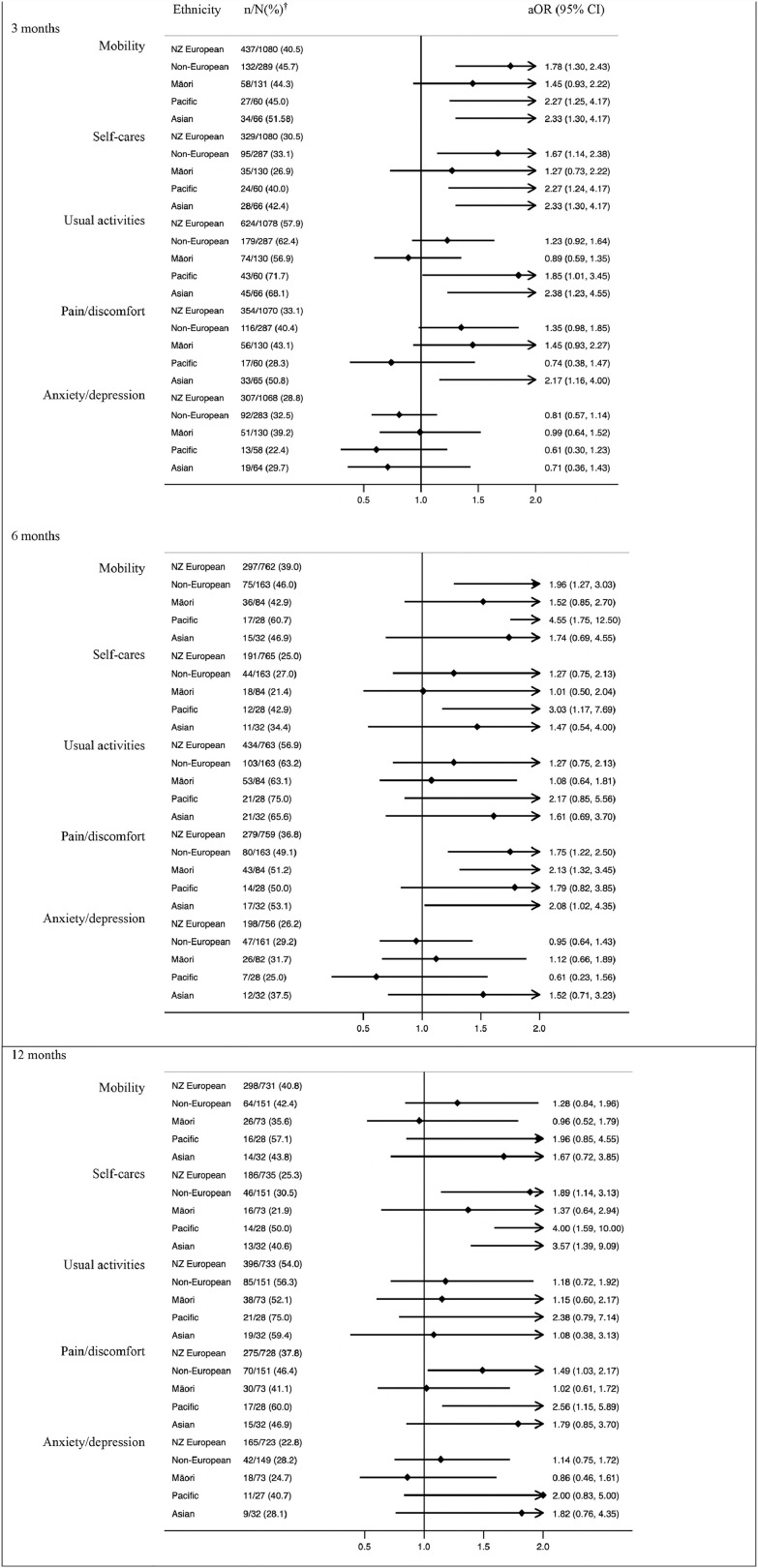
Quality of life (EQ-5D-3L) – reporting any problems. ^†^n/N(%): the numerator refers to the number of people who achieved an outcome and the denominator refers to the number of people for whom data was available had data available.

For quality of life, non-Europeans reported problems with mobility, self-care tasks and pain more often than Europeans during the twelve-month follow-up period (Figure 4). There were no ethnic differences in reports of difficulties with usual activities or in experiencing anxiety and/or depression at any of the three time points, except for Asian patients who reported more difficulties with usual activities at 3 months (aOR=2·38 (1·23-4·55). There were some ethnic differences in reported health state (EQ-VAS) between European New Zealanders and Pacific and Asian patients (Table 2). However, when comparing non-Europeans with European New Zealanders at each time point there was no difference in reported health state.

**Table 2:** EQ-VAS by ethnicity.

	NZ European	Māori	Pacific	Asian	Other
VAS 3 months Median (IQR)	75 (50-85)	75 (50-85)	76 (50-89)	65 (50-80)*	75 (50-80)
VAS 6 months Median (IQR)	75 (60-85)	75 (50-80)	50 (47·5-80)*	75 (50-90)	80 (65-85)
VAS 12 months Median (IQR)	75 (60-85)	75 (55-85)	75 (50-89)	75 (60-90)	80·5 (55-85)

⁎ p<0.05 for different EQ-VAS (reference=NZ European)

## Discussion

We present new evidence on the association between ethnicity and stroke care access and post-stroke outcomes from a prospective nationwide study of all admitted patients with stroke covering the entire stroke care continuum up to 12 months. We found worse functional outcomes for non-Europeans at three, six and 12 months following stroke. Specifically, Māori and Pacific peoples experienced poorer outcomes at three and 12 months and Māori were more likely to have died by 12 months.

The majority of explored interventions were accessed equally across all ethnicities providing reassurance that high quality and equal access across ethnicities is achievable and already in place for many aspects of stroke care in New Zealand. Reperfusion therapy access for those who were eligible was similar across ethnic groups, something we showed previously.^14^ This contrasts with other international studies that have found ethnic disparities in reperfusion therapy access,^15^^,^^16^ and which have proposed that poor awareness and long presentation delays could account for this observation.^16^^,^^17^ In this study, there was no difference in the proportion of non-Europeans compared with Europeans that presented to hospital within four hours of symptom onset. This may in part be attributable to New Zealand's public awareness FAST (“Face, Arm, Speech, Time”) campaigns, which have focussed on high-risk populations.^18^ Similarly, rehabilitation was generally accessed equally across ethnic groups, consistent with recent international work.^19^ It is encouraging to see that non-Europeans may be prioritised for more frequent post-discharge community team contacts.

However, several post-stroke interventions were not equally accessed by the different ethnic groups. Non-Europeans had poorer access to ASU care and worse access to early swallow assessments and a strong trend toward a lower rate of early mobilisation. Also, the trend toward a lower likelihood of accessing at least one hour of physical therapy in non-Europeans in inpatient rehabilitation deserves further exploration. We have previously shown that patients presenting to urban hospitals generally experience better access to key stroke^20^ interventions. While the analyses were adjusted for rurality and hospital in a multi-level mixed effects model it is possible that a degree of residual confounding could explain some of the disparities especially between minority ethnic groups^20^ as Pacific peoples and Asians tend to live in urban centres while Māori more commonly reside rurally. For example, the trend toward poorer DVT prophylaxis in Māori and greater access to telemetry in Asians may in part be attributable to these geographic factors. The overall greater access to CT by non-Europeans almost certainly relates to age, as the only reason patients with stroke do not undergo CT scanning is early palliation for severe stroke in frail people of older age, who are predominantly European. However, it is difficult to reconcile the overall lower access to MRI scans by all minority populations. A possible explanation is that these groups who, on average, presented with more severe and thus likely clearer definite clinical or CT evidence of stroke, potentially did not require MRI imaging in the context of relatively low rate of MRI scanning in New Zealand. However, other ethnicity-based factors around disparate MRI access cannot be excluded and should undergo further investigation. Thus, we have identified several areas within acute and rehabilitation stroke care that should be further explored to enhance equity of access to best practice stroke care for non-Europeans and represent additional priority improvement areas to help drive equality in patient outcomes across all ethnic groups.

Despite adjusting the analysis for stroke risk factors and baseline disability, some of the outcome differences are probably still influenced by poorer underlying health status and we should not dismiss the importance of risk factor management. It is encouraging that this study found increased emphasis being placed on exercise, diet, and stroke symptom education offered to non-Europeans, and generally good implementation of secondary prevention medications. It is noteworthy that it is particularly Pacific peoples who are accessing enhanced prevention counselling, potentially facilitated through better access to stroke clinical nurse specialists while in the hospital. Despite controlling for hospital in the analysis, it is likely that this disparity is driven by two urban stroke services that manage most Pacific patients across the country and have a large proportion of Pacific nurses.

By comparison, Māori appear to receive less focussed prevention services, further supported by a lower rate of appropriate anticoagulation prescriptions, despite what otherwise appears as ‘equal’ prevention access. This underscores the important difference between ‘equality’ and ‘equity,’ where Māori almost certainly require not only equal but enhanced access compared with European New Zealanders to achieve true equity in access and equality in outcome. Health and stroke disparities between Indigenous and other ethnic minority peoples have been described internationally^15^^,^^21^^,^^22^ and underlying causes for the gaps, including colonisation, intergenerational trauma and institutionalised racism, are well described.^23^, ^24^, ^25^ Despite the broadly available cultural support services for Māori (as well as Pacific peoples) these were inconsistently offered by stroke teams and accessed by only a minority of patients. Enhancing cultural awareness and competence and aiming for greater diversity among the stroke workforce to improve culturally appropriate care represents another area for improvement.^26^ Recent health system reforms in New Zealand, including the establishment of a Māori Health Authority to monitor the whole of health system, support cultural safety and resource Indigenous innovation,^27^ such as that of the Take Charge intervention for Māori and Pacific peoples with stroke,^28^ are promising.

A strength of this study is the prospective consecutive patient recruitment from all acute stroke hospitals in New Zealand achieving a pre-determined sample size, giving excellent representation and minimal risk of selection bias. Previous New Zealand studies exploring stroke and ethnicity have been largely retrospective, limited to a single geographic area, relied purely on health administrative data, and/or have had a smaller sample size.^8^, ^9^, ^10^, ^11^ Further strengths of this study include data collection across the care continuum including community care, enhancing the ability to explore and control of multiple interventions across the patient journey, and patient follow-up to twelve months.

This study has several limitations. It was powered primarily to detect disparities in outcomes among non-European New Zealanders collectively and the sub-group of the Indigenous Māori. Sample sizes for the other ethnicities were smaller. In addition, due to multiple comparisons it is possible that some significant and especially non-significant trend results are chance occurrences. Therefore, results for ethnic sub-groups, especially Pacific peoples and Asians and non-significant trends generally should be interpreted with caution and viewed primarily as exploratory. Despite adjusting results for established strong predictors of stroke outcomes and differences in baseline characteristics, residual confounding cannot be excluded. While follow-up rates were generally satisfactory, the finding that those who were not followed up had more severe strokes and risk factors, combined with lower follow-up rates for non-Europeans may have contributed to some bias. However, this would have resulted in an underestimation (rather than overestimation) of the poorer outcomes reported for non-Europeans making it likely that our overall conclusions remain valid, although the magnitude of disparity may be somewhat greater than what we report. Finally, as the study is entirely New Zealand based, results may not be generalisable to other – especially predominantly non-European - populations.

In conclusion, this study found poorer stroke outcomes in non-Europeans including greater mortality with evidence for unequal access to some key acute and rehabilitation best practice stroke interventions especially for Māori. While some of these access differences may, in part, be due to poorer non-urban service access, risk factors and residual confounding, these were controlled for and there is evidence of residual disparity that cannot be solely explained by these factors, which we speculate may be related to institutional racism, as also shown by others.^29^^,^^30^ It is encouraging to see that the majority of key interventions were accessed equally and that especially Pacific peoples are receiving enhanced post-stroke risk factor counselling. Replicating this for Māori should be a priority and the establishment of the New Zealand Māori Health Authority is an important step toward achieving this goal. Increasing awareness and competence on the part of stroke healthcare providers in offering culturally safe and concordant patient treatment and support could further enhance progress. This is often best achieved through recruitment of an ethnically diverse workforce with a specific emphasis on Māori health provider capacity building.

## Data sharing

De-identified individual participant data, data dictionary, protocol, and consent forms can be requested via the corresponding author and will be available once all results from the study have been published assuming appropriate ethics approval is achieved.

## Contributiors

Stephanie G Thompson - data collection, data analysis, writing: original draft; accessed and verified the underlying data

P Alan Barber - funding acquisition, investigation methodology, writing: review and editing

John Gommans - funding acquisition, investigation methodology, writing: review and editing

Dominique A Cadilhac - funding acquisition, investigation methodology, writing: review and editing

Alan Davis - funding acquisition, investigation methodology, writing: review and editing

John N Fink - funding acquisition, investigation methodology, writing: review and editing

Matire Harwood - funding acquisition, investigation methodology, writing: review and editing

William Levack - writing: review and editing

Harry McNaughton - funding acquisition, investigation methodology, writing: review and editing

Valery L Feigin - funding acquisition, investigation methodology, writing: review and editing

Virginia Abernethy - funding acquisition, investigation methodology, writing: review and editing

Jackie Girvan - funding acquisition, investigation methodology, writing: review and editing

Hayley Denison - investigation methodology

Marine Corbin - writing: review and editing

Andrew Wilson - writing: review and editing

Jeroen Douwes - funding acquisition, investigation methodology, writing: review and editing

Annemarei Ranta - conceptualisation, funding acquisition, investigation methodology, data analysis, writing: review and editing, accessed and verified the underlying data

## Declaration of interests

All authors have completed the ICMJE uniform disclosure form at www.icmje.org/coi_disclosure.pdf and declare: no disclosures, except for DC, JD and JK who report grants from the Health Research Council of New Zealand during the conduct of the study; DC also reports grants from National Health and Medical Research Council, grants from Medtronic, grants from Amgen, grants from Stroke Foundation, grants from Academy of Science, grants from Heart Foundation, grants from CSIRO, grants from Victorian Agency for Health Innovation, grants from Boehringer Ingelheim, grants from Melbourne Health, grants from National Institute for Health Research UK, grants from Western Australian government, grants from South Australian government, outside the submitted work. MH reports an Appointed member of Waitemata District Health Board 2016 to 2019; no other relationships or activities that could appear to have influenced the submitted work.

## Appendix Supplementary materials

Image, application 1

Image, application 2

Image, application 3

Image, application 4

Image, application 5
