# Supplementary material for: The impact of ethnicity on stroke care access and patient outcomes: a New Zealand nationwide observational study
Source: Lancet Reg Health West Pac. 2022 Jan 3;20:100358. doi: 10.1016/j.lanwpc.2021.100358 (PMC8743211; doi:10.1016/j.lanwpc.2021.100358)
Supplement: Supplementary file 1 [file mmc1.docx]

**Supplementary Table 1: Follow-up rates by ethnicity**

|  | **European** | **Māori** | **Pacific** | **Asian** | **Other** | **p-value** |
| --- | --- | --- | --- | --- | --- | --- |
| **Total study cohort followed to 3 months** | **N=1823**  **n (%)** | **N=273**  **n (%)** | **N=114**  **n (%)** | **N=115**  **n (%)** | **N=54**  **n (%)** |  |
| **3 months post-stroke** |  |  |  |  |  | <0·001 |
| **Outcomes at follow-up** | 1470 (81) | 185 (68) | 71 (62) | 83 (72) | 44 (81) |  |
| **Lost to follow-up** | 353 (19) | 88 (32) | 43 (38) | 32 (28) | 10 (19) |  |
|  | **European** | **Māori** | **Pacific** | **Asian** | **Other** | **p-value** |
| **Extended follow-up cohort*** | **N=1216**  **n (%)** | **N=168**  **n (%)** | **N=51**  **n (%)** | **N=58**  **n (%)** | **N=33**  **n (%)** |  |
| **3 months post-stroke** |  |  |  |  |  | 0·05 |
| **Outcomes at follow-up** | 1215 (100) | 166 (99) | 51 (100) | 58 (100) | 33 (100) |  |
| **6 months post-stroke** |  |  |  |  |  | <0·001 |
| **Outcomes at follow-up** | 1179 (97) | 143 (85) | 45 (88) | 53 (91) | 31 (94) |  |
| **Lost to follow-up** | 37 (3) | 25 (15) | 6 (12) | 5 (9) | 2 (6) |  |
| **12 months post-stroke** |  |  |  |  |  | <0·001 |
| **Outcomes at follow-up** | 1153 (95) | 133 (79) | 41 (80) | 50 (86) | 31 (94) |  |
| **Lost to follow-up** | 63 (5) | 35 (21) | 10 (20) | 8 (14) | 2 (6) |  |

*A subset of patients sequentially recruited at 3 months for extended follow-up at 6 and 12 months and data linkage.
