# Supplementary material for: The impact of ethnicity on stroke care access and patient outcomes: a New Zealand nationwide observational study
Source: Lancet Reg Health West Pac. 2022 Jan 3;20:100358. doi: 10.1016/j.lanwpc.2021.100358 (PMC8743211; doi:10.1016/j.lanwpc.2021.100358)
Supplement: Supplementary file 2 [file mmc2.docx]

**Supplementary Table 2: Baseline characteristics of patients with and without follow-up data for the total study cohort**

|  | **Follow-up data available** | **Lost to follow-up** | **p-value** |
| --- | --- | --- | --- |
| **3 months post-stroke** | **N=1853**  **n (%)** | **N=526**  **n (%)** |  |
| Age (median years, IQR) | 78 (68 - 86) | 73 (62 - 83) | <0·001 |
| Sex | 895 (48) | 265 (50) | 0·40 |
| Type of stroke |  |  | <0·001 |
| Intracerebral haemorrhage | 225 (12) | 66 (12) |  |
| Cerebral infarction | 1527 (82) | 410 (78) |  |
| Stroke unspecified* | 101 (6) | 50 (10) |  |
| Stroke severity |  |  |  |
| GCS verbal <5 | 1153 (62) | 363 (69) | 0·003 |
| Requires assistance to walk | 808 (44) | 232 (44) | 0·82 |
| Arms MRC <3/5 | 1145 (62) | 356 (68) | 0·01 |
| Independent (mRS pre-stroke) | 1588 (87) | 452 (87) | 0·69 |
| Glucose (median years, IQR) | 7·1 (5·9 - 8·8) | 7 (6·1 - 8·8) | 0·67 |
| SBP (median years, IQR) | 161 (140 - 183) | 157.5 (137 - 180) | 0·09 |
| Comorbidities |  |  |  |
| Hypertension | 1318 (71) | 377 (72) | 0·81 |
| Diabetes | 433 (23) | 138 (26) | 0·17 |
| Dyslipidaemia | 773 (42) | 225 (43) | 0·66 |
| Atrial fibrillation | 642 (35) | 165 (31) | 0·16 |
| Smoking | 203 (11) | 84 (16) | 0·002 |
| Urban hospital | 1136 (61) | 291 (55) | 0·01 |
| Ethnicity |  |  | <0·001 |
| European | 1470 (79) | 353 (67) |  |
| Maori | 185 (10) | 88 (17) |  |
| Pacific | 71 (4) | 43 (8) |  |
| Asian | 83 (4) | 32 (6) |  |
| Other/unknown | 44 (2) | 10 (2) |  |
| **6 months post-stroke** | **N=1451**  **n (%)** | **N=75**  **n (%)** |  |
| Age (median years, IQR) | 79 (69 - 86) | 65 (53 - 80) | <0·001 |
| Sex | 695 (48) | 37 (49) | 0·81 |
| Type of stroke |  |  | 0·99 |
| Intracerebral haemorrhage | 182 (13) | 9 (12) |  |
| Cerebral infarction | 1192 (82) | 62 (83) |  |
| Stroke unspecified | 77 (5) | 4 (5) |  |
| Stroke severity |  |  |  |
| GCS verbal <5 | 890 (61) | 53 (71) | 0·11 |
| Requires assistance to walk | 615 (43) | 39 (52) | 0·11 |
| Arms MRC <3/5 | 866 (60) | 53 (71) | 0·06 |
| Independent (mRS pre-stroke) | 1246 (87) | 68 (91) | 0·32 |
| Glucose (median years, IQR) | 7·1 (5·9 - 8·8) | 7·1 (5·8 - 8·7) | 0·93 |
| SBP (median years, IQR) | 160 (140 - 182) | 150 (132 - 174) | 0·02 |
| Comorbidities |  |  |  |
| Hypertension | 1031 (71) | 49 (65) | 0·29 |
| Diabetes | 315 (22) | 27 (36) | 0·004 |
| Dyslipidaemia | 589 (41) | 34 (45) | 0·42 |
| Atrial fibrillation | 506 (35) | 19 (25) | 0·09 |
| Smoking | 138 (10) | 32 (43) | <0·001 |
| Urban hospital | 877 (60) | 37 (49) | 0·06 |
| Ethnicity |  |  | <0·001 |
| European | 1179 (81) | 37 (49) |  |
| Maori | 143 (10) | 25 (33) |  |
| Pacific | 45 (3) | 6 (8) |  |
| Asian | 53 (4) | 5 (7) |  |
| Other/unknown | 31 (2) | 2 (3) |  |
| **12 months post-stroke** | **N=1408**  **n (%)** | **N=118**  **n (%)** |  |
| Age (median years, IQR) | 79 (69 - 86) | 67 (57 - 80) | <0·001 |
| Sex | 673 (48) | 59 (50) | 0·65 |
| Type of stroke |  |  | 0·72 |
| Intracerebral haemorrhage | 179 (13) | 12 (10) |  |
| Cerebral infarction | 1154 (82) | 100 (85) |  |
| Stroke unspecified | 75 (5) | 6 (5) |  |
| Stroke severity |  |  |  |
| GCS verbal <5 | 857 (61) | 86 (73) | 0·01 |
| Requires assistance to walk | 590 (42) | 64 (54) | 0·01 |
| Arms MRC <3/5 | 834 (59) | 85 (72) | 0·01 |
| Independent (mRS pre-stroke) | 1208 (87) | 106 (90) | 0·33 |
| Glucose (median years, IQR) | 7·1 (5·9 - 8·8) | 7·1 (5·6 - 8·7) | 0·73 |
| SBP (median years, IQR) | 161 (140 - 183) | 150 (134·5 - 176·5) | 0·01 |
| Comorbidities |  |  |  |
| Hypertension | 998 (71) | 82 (69) | 0·75 |
| Diabetes | 301 (21) | 41 (35) | 0·001 |
| Dyslipidaemia | 575 (41) | 48 (41) | 0·97 |
| Atrial fibrillation | 490 (35) | 35 (30) | 0·26 |
| Smoking | 127 (9) | 43 (36) | <0·001 |
| Urban hospital | 845 (60) | 69 (58) | 0·74 |
| Ethnicity |  |  | <0·001 |
| European | 1153 (82) | 63 (53) |  |
| Maori | 133 (9) | 35 (30) |  |
| Pacific | 41 (3) | 10 (8) |  |
| Asian | 50 (4) | 8 (7) |  |
| Other/unknown | 31 (2) | 2 (2) |  |

*includes stroke unspecified and those of unknown cause.
