# Supplementary material for: The impact of ethnicity on stroke care access and patient outcomes: a New Zealand nationwide observational study
Source: Lancet Reg Health West Pac. 2022 Jan 3;20:100358. doi: 10.1016/j.lanwpc.2021.100358 (PMC8743211; doi:10.1016/j.lanwpc.2021.100358)
Supplement: Supplementary file 3 [file mmc3.pdf]

Supplementary Figure 1: Access to stroke interventions/care by individual ethnic groups

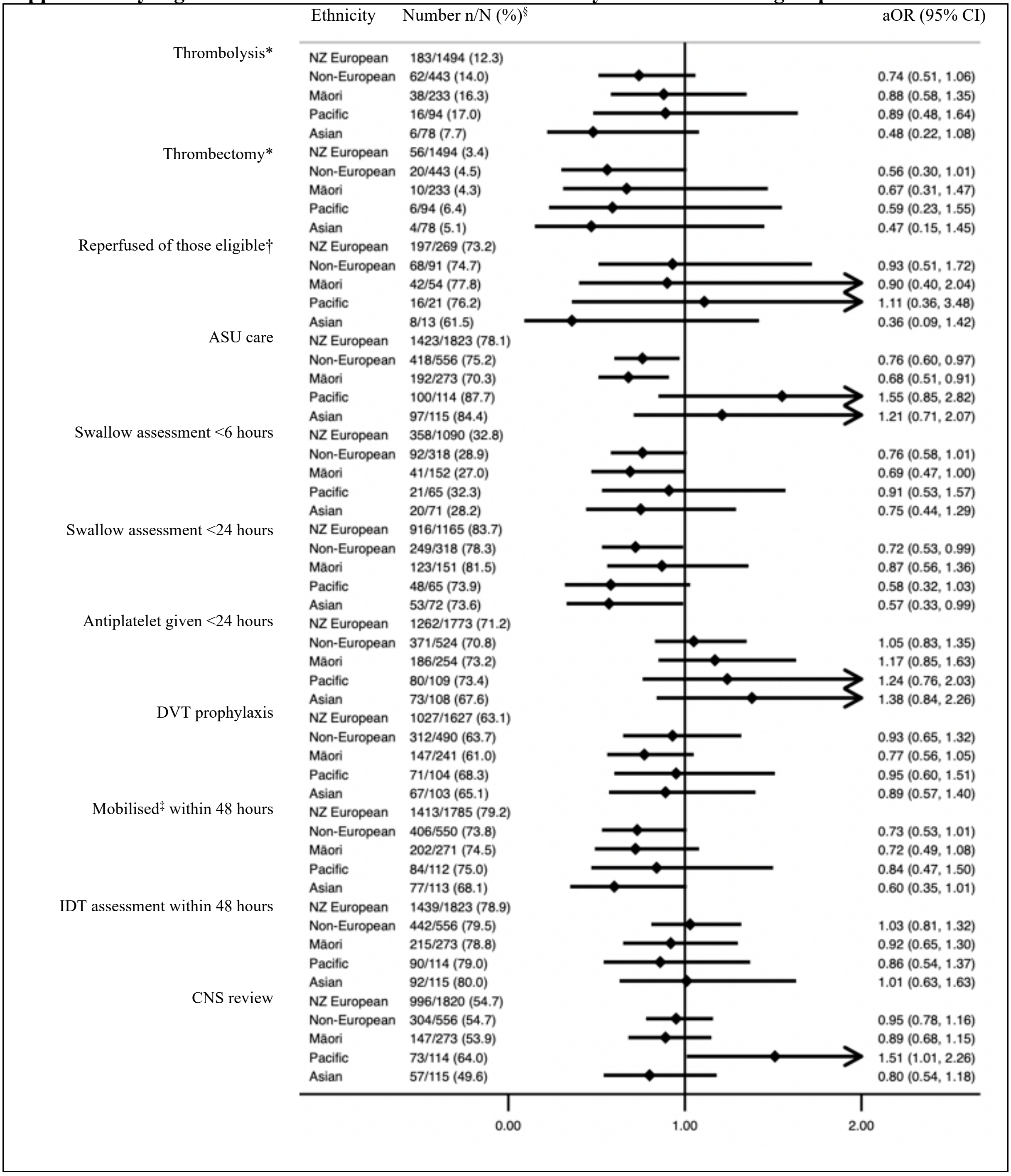

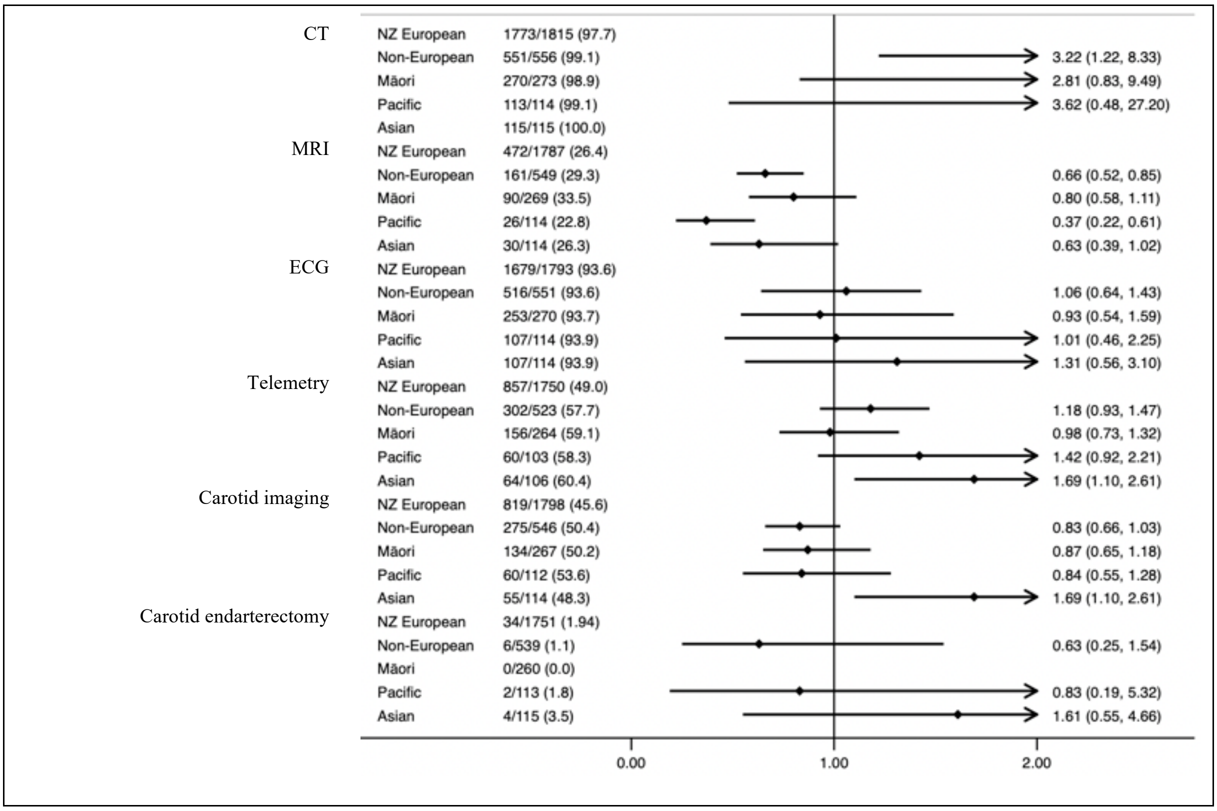
CT
NZ European 1773/1815 (97.7)
Non-European 551/556 (99.1) 3.22 (1.22, 8.33)
Māori 270/273 (98.9) 2.81 (0.83, 9.49)
Pacific 113/114 (99.1) 3.62 (0.48, 27.20)
Asian 115/115 (100.0)
MRI
NZ European 472/1787 (26.4)
Non-European 161/549 (29.3) 0.66 (0.52, 0.85)
Māori 90/269 (33.5) 0.80 (0.58, 1.11)
Pacific 26/114 (22.8) 0.37 (0.22, 0.61)
Asian 30/114 (26.3) 0.63 (0.39, 1.02)
ECG
NZ European 1679/1793 (93.6)
Non-European 516/551 (93.6) 1.06 (0.64, 1.43)
Māori 253/270 (93.7) 0.93 (0.54, 1.59)
Pacific 107/114 (93.9) 1.01 (0.46, 2.25)
Asian 107/114 (93.9) 1.31 (0.56, 3.10)
Telemetry
NZ European 857/1750 (49.0)
Non-European 302/523 (57.7) 1.18 (0.93, 1.47)
Māori 156/264 (59.1) 0.98 (0.73, 1.32)
Pacific 60/103 (58.3) 1.42 (0.92, 2.21)
Asian 64/106 (60.4) 1.69 (1.10, 2.61)
Carotid imaging
NZ European 819/1798 (45.6)
Non-European 275/546 (50.4) 0.83 (0.66, 1.03)
Māori 134/267 (50.2) 0.87 (0.65, 1.18)
Pacific 60/112 (53.6) 0.84 (0.55, 1.28)
Asian 55/114 (48.3) 1.69 (1.10, 2.61)
Carotid endarterectomy
NZ European 34/1751 (1.94)
Non-European 6/539 (1.1) 0.63 (0.25, 1.54)
Māori 0/260 (0.0)
Pacific 2/113 (1.8) 0.83 (0.19, 5.32)
Asian 4/115 (3.5) 1.61 (0.55, 4.66)
0.00
1.00
2.00

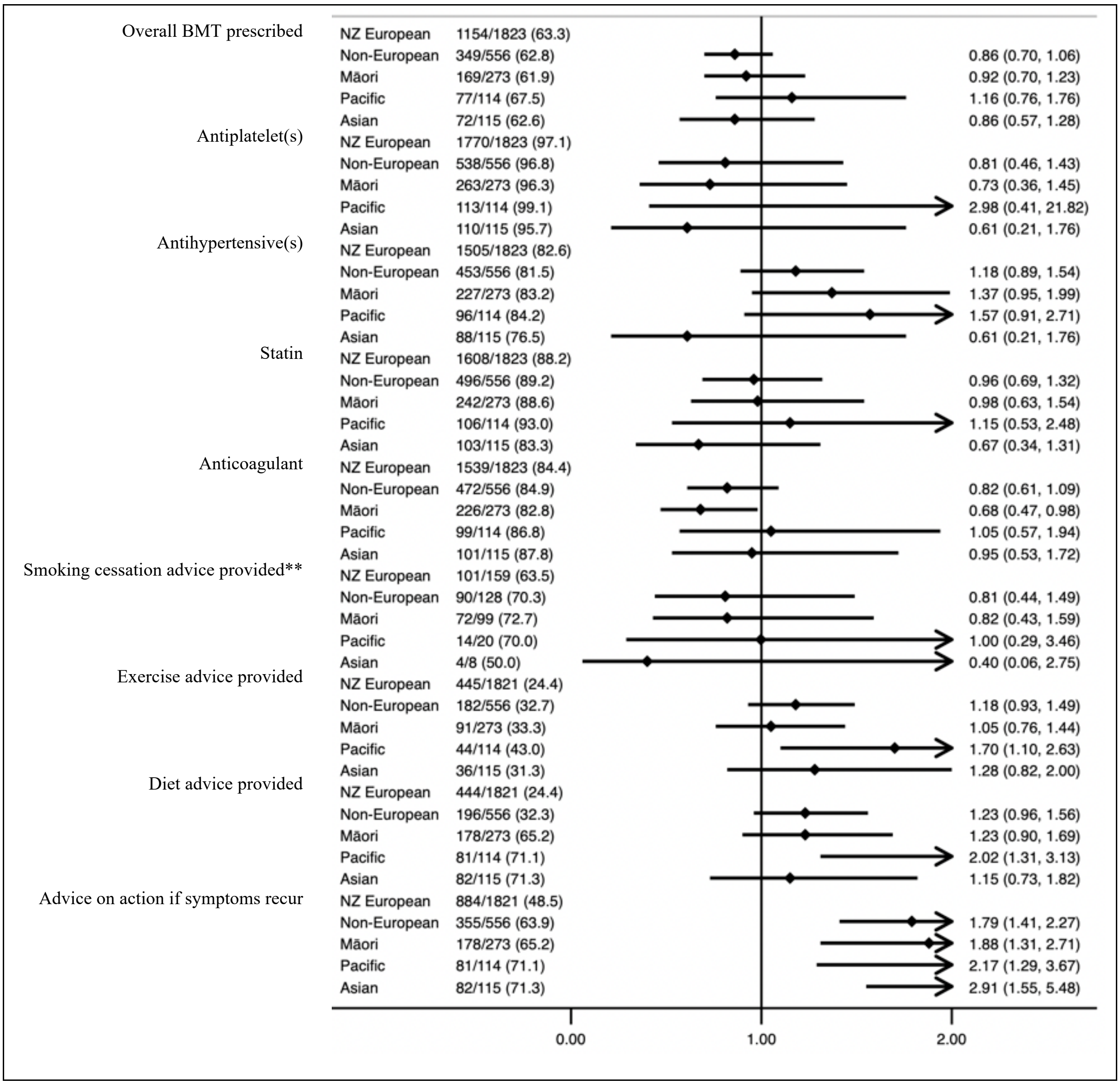

Overall BMT prescribed
NZ European 1154/1823 (63.3)
Non-European 349/556 (62.8) 0.86 (0.70, 1.06)
Māori 169/273 (61.9) 0.92 (0.70, 1.23)
Pacific 77/114 (67.5) 1.16 (0.76, 1.76)
Asian 72/115 (62.6) 0.86 (0.57, 1.28)
Antiplatelet(s)
NZ European 1770/1823 (97.1)
Non-European 538/556 (96.8) 0.81 (0.46, 1.43)
Māori 263/273 (96.3) 0.73 (0.36, 1.45)
Pacific 113/114 (99.1) 2.98 (0.41, 21.82)
Asian 110/115 (95.7) 0.61 (0.21, 1.76)
Antihypertensive(s)
NZ European 1505/1823 (82.6)
Non-European 453/556 (81.5) 1.18 (0.89, 1.54)
Māori 227/273 (83.2) 1.37 (0.95, 1.99)
Pacific 96/114 (84.2) 1.57 (0.91, 2.71)
Asian 88/115 (76.5) 0.61 (0.21, 1.76)
Statin
NZ European 1608/1823 (88.2)
Non-European 496/556 (89.2) 0.96 (0.69, 1.32)
Māori 242/273 (88.6) 0.98 (0.63, 1.54)
Pacific 106/114 (93.0) 1.15 (0.53, 2.48)
Asian 103/115 (83.3) 0.67 (0.34, 1.31)
Anticoagulant
NZ European 1539/1823 (84.4)
Non-European 472/556 (84.9) 0.82 (0.61, 1.09)
Māori 226/273 (82.8) 0.68 (0.47, 0.98)
Pacific 99/114 (86.8) 1.05 (0.57, 1.94)
Asian 101/115 (87.8) 0.95 (0.53, 1.72)
Smoking cessation advice provided**
NZ European 101/159 (63.5)
Non-European 90/128 (70.3) 0.81 (0.44, 1.49)
Māori 72/99 (72.7) 0.82 (0.43, 1.59)
Pacific 14/20 (70.0) 1.00 (0.29, 3.46)
Asian 4/8 (50.0) 0.40 (0.06, 2.75)
Exercise advice provided
NZ European 445/1821 (24.4)
Non-European 182/556 (32.7) 1.18 (0.93, 1.49)
Māori 91/273 (33.3) 1.05 (0.76, 1.44)
Pacific 44/114 (43.0) 1.70 (1.10, 2.63)
Asian 36/115 (31.3) 1.28 (0.82, 2.00)
Diet advice provided
NZ European 444/1821 (24.4)
Non-European 196/556 (32.3) 1.23 (0.96, 1.56)
Māori 178/273 (65.2) 1.23 (0.90, 1.69)
Pacific 81/114 (71.1) 2.02 (1.31, 3.13)
Asian 82/115 (71.3) 1.15 (0.73, 1.82)
Advice on action if symptoms recur
NZ European 884/1821 (48.5)
Non-European 355/556 (63.9) 1.79 (1.41, 2.27)
Māori 178/273 (65.2) 1.88 (1.31, 2.71)
Pacific 81/114 (71.1) 2.17 (1.29, 3.67)
Asian 82/115 (71.3) 2.91 (1.55, 5.48)
0.00
1.00
2.00

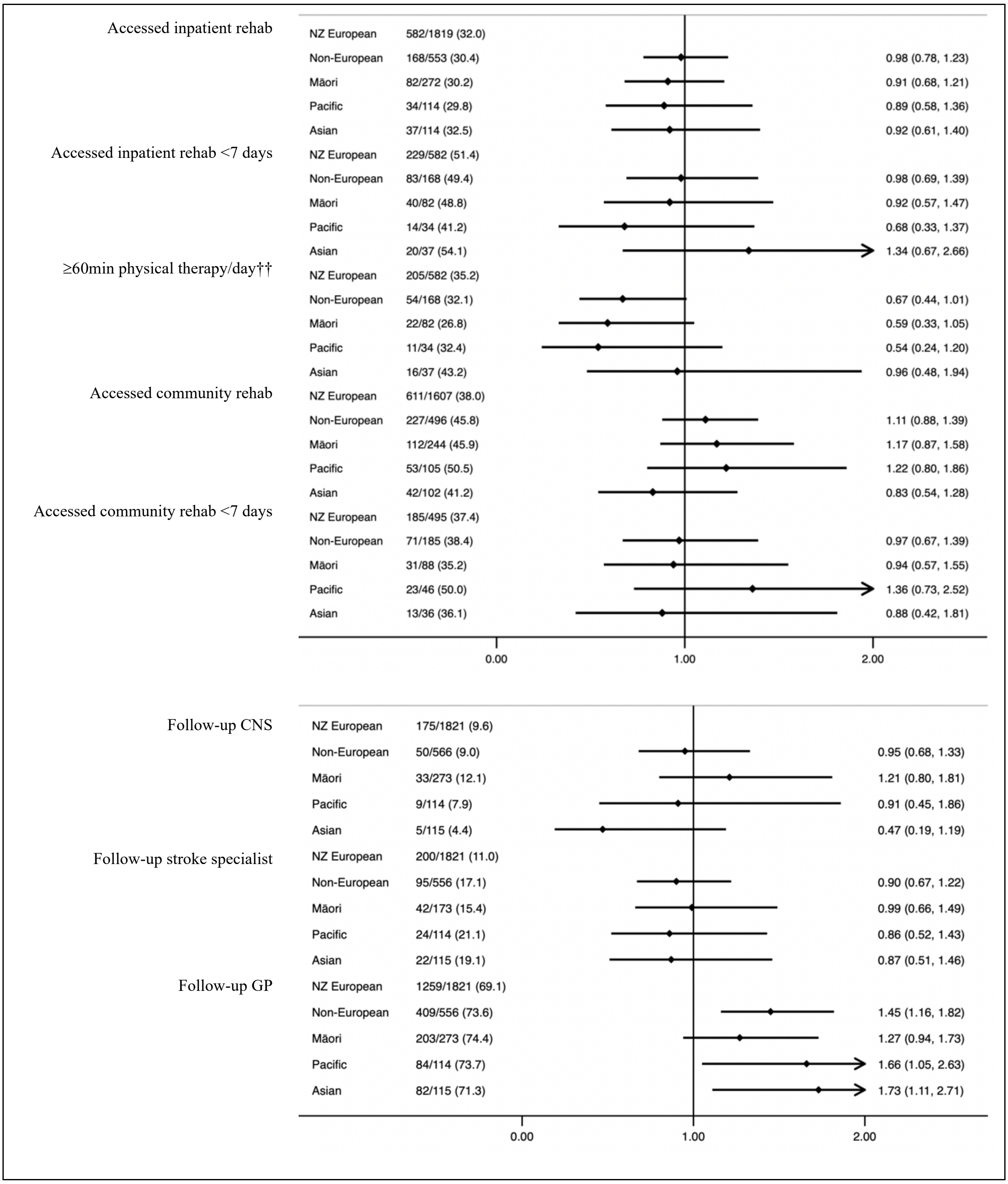
Accessed inpatient rehab
NZ European 582/1819 (32.0)
Non-European 168/553 (30.4) 0.98 (0.78, 1.23)
Māori 82/272 (30.2) 0.91 (0.68, 1.21)
Pacific 34/114 (29.8) 0.89 (0.58, 1.36)
Asian 37/114 (32.5) 0.92 (0.61, 1.40)
Accessed inpatient rehab <7 days
NZ European 229/582 (51.4)
Non-European 83/168 (49.4) 0.98 (0.69, 1.39)
Māori 40/82 (48.8) 0.92 (0.57, 1.47)
Pacific 14/34 (41.2) 0.68 (0.33, 1.37)
Asian 20/37 (54.1) 1.34 (0.67, 2.66)
≥60min physical therapy/day††
NZ European 205/582 (35.2)
Non-European 54/168 (32.1) 0.67 (0.44, 1.01)
Māori 22/82 (26.8) 0.59 (0.33, 1.05)
Pacific 11/34 (32.4) 0.54 (0.24, 1.20)
Asian 16/37 (43.2) 0.96 (0.48, 1.94)
Accessed community rehab
NZ European 611/1607 (38.0)
Non-European 227/496 (45.8) 1.11 (0.88, 1.39)
Māori 112/244 (45.9) 1.17 (0.87, 1.58)
Pacific 53/105 (50.5) 1.22 (0.80, 1.86)
Asian 42/102 (41.2) 0.83 (0.54, 1.28)
Accessed community rehab <7 days
NZ European 185/495 (37.4)
Non-European 71/185 (38.4) 0.97 (0.67, 1.39)
Māori 31/88 (35.2) 0.94 (0.57, 1.55)
Pacific 23/46 (50.0) 1.36 (0.73, 2.52)
Asian 13/36 (36.1) 0.88 (0.42, 1.81)
0.00 1.00 2.00
Follow-up CNS
NZ European 175/1821 (9.6)
Non-European 50/566 (9.0) 0.95 (0.68, 1.33)
Māori 33/273 (12.1) 1.21 (0.80, 1.81)
Pacific 9/114 (7.9) 0.91 (0.45, 1.86)
Asian 5/115 (4.4) 0.47 (0.19, 1.19)
Follow-up stroke specialist
NZ European 200/1821 (11.0)
Non-European 95/556 (17.1) 0.90 (0.67, 1.22)
Māori 42/173 (15.4) 0.99 (0.66, 1.49)
Pacific 24/114 (21.1) 0.86 (0.52, 1.43)
Asian 22/115 (19.1) 0.87 (0.51, 1.46)
Follow-up GP
NZ European 1259/1821 (69.1)
Non-European 409/556 (73.6) 1.45 (1.16, 1.82)
Māori 203/273 (74.4) 1.27 (0.94, 1.73)
Pacific 84/114 (73.7) 1.66 (1.05, 2.63)
Asian 82/115 (71.3) 1.73 (1.11, 2.71)
0.00 1.00 2.00

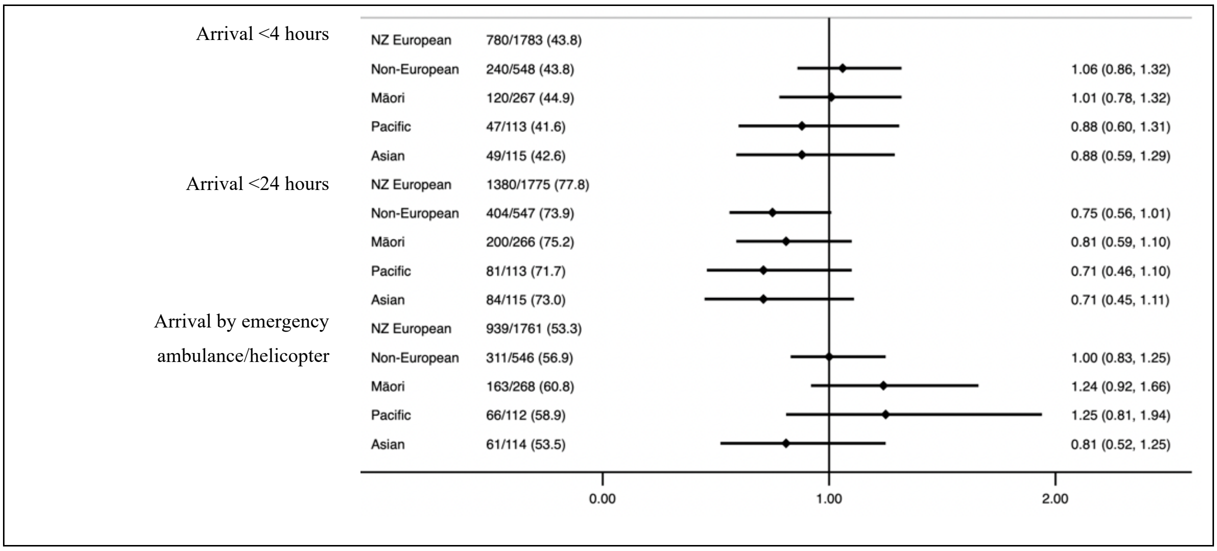

aOR=adjusted odds ratio (all outcomes were adjusted for pre-morbid level of function, age sex, rurality, stroke severity, baseline characteristic differences of p<0.1, and intervention specific covariates such as time delay to reach hospital, mode of transport for reperfusion therapies and palliation within 24 hours for early mobilisation and allied health input. Covariates were backward eliminated unless removal substantially impacted odds ration aiming to minimise number of covariates and optimise model fit); 95% CI=95% confidence interval; ASU=acute stroke unit; DVT=deep vein thrombosis; IDT=interdisciplinary team; BMT=best medical therapy; CNS review refers to a stroke clinical nurse specialist review of the patient on the ward while an inpatient; BMT='best medical therapy' refers to antiplatelet(s), statins, and anti-hypertensives for non-cardioembolic ischaemic stroke patients, anti-hypertensives for ICH patients attributed to hypertension, and anticoagulation for patients with cardioembolic stroke unless any contraindications documented; GP=general practitioner; Follow-up with stroke nurse refers to post-discharge follow-up appointment with a stroke clinical nurse specialist; § n/N(%): the numerator refers to the number of people that received the intervention and the denominator to the number of people for whom we had data available; *Denominator for these analyses consists of only those patients with a primary diagnosis of ischaemic stroke; †'Reperfused of those eligible' refers to patients undergoing thrombolysis and/or thrombectomy among those who presented within the require time window and did not have appropriate exclusion criteria; ‡'Mobilised' refers to any 'out of bed activity';**Analysis limited to current smokers at the time of presentation; ††weekdays only.
