# Supplementary material for: The impact of ethnicity on stroke care access and patient outcomes: a New Zealand nationwide observational study
Source: Lancet Reg Health West Pac. 2022 Jan 3;20:100358. doi: 10.1016/j.lanwpc.2021.100358 (PMC8743211; doi:10.1016/j.lanwpc.2021.100358)
Supplement: Supplementary file 4 [file mmc4.docx]

**Supplementary Table 3: Baseline characteristics of patients with and without follow-up data for the European cohort**

|  | **Outcomes at follow-up** | **Lost to follow-up** | **p-value** |
| --- | --- | --- | --- |
| **3 months post-stroke** | **N=1470**  **n (%)** | **N=353**  **n (%)** |  |
| Age (median years, IQR) | 80 (71 - 87) | 78 (68 - 86) | 0·02 |
| Sex | 698 (47) | 180 (51) | 0·24 |
| Type of stroke |  |  | <0·001 |
| Intracerebral haemorrhage | 168 (11) | 41 (12) |  |
| Cerebral infarction | 1225 (84) | 269 (76) |  |
| Stroke unspecified | 77 (5) | 43 (12) |  |
| Stroke severity |  |  |  |
| GCS verbal <5 | 937 (64) | 243 (69) | 0·06 |
| Requires assistance to walk | 655 (45) | 150 (43) | 0·50 |
| Arms MRC <3/5 | 930 (63) | 247 (70) | 0·02 |
| Independent (mRS pre-stroke) | 1249 (86) | 291 (83) | 0·23 |
| Glucose (median years, IQR) | 7 (5·9 - 8·7) | 7 (6 - 8·6) | 0·995 |
| SBP (median years, IQR) | 163 (140 - 185) | 157 (139 - 182) | 0·14 |
| Comorbidities |  |  |  |
| Hypertension | 1042 (71) | 252 (71) | 0·85 |
| Diabetes | 288 (20) | 71 (20) | 0·83 |
| Dyslipidaemia | 596 (41) | 149 (42) | 0·57 |
| Atrial fibrillation | 524 (36) | 114 (32) | 0·24 |
| Smoking | 126 (9) | 33 (9) | 0·64 |
| Urban hospital | 878 (60) | 185 (52) | 0·01 |
| **6 months post-stroke** | **N=1179**  **n (%)** | **N=37**  **n (%)** |  |
| Age (median years, IQR) | 80 (71 - 87) | 88 (88 - 88) | 0·49 |
| Sex | 569 (47) | 0 (0) | 0·66 |
| Type of stroke |  |  | 0·82 |
| Intracerebral haemorrhage | 143 (12) | 0 (0) |  |
| Cerebral infarction | 1013 (84) | 1 (100) |  |
| Stroke unspecified | 57 (5) | 0 (0) |  |
| Stroke severity |  |  |  |
| GCS verbal <5 | 772 (64) | 1 (100) | 0·88 |
| Requires assistance to walk | 533 (44) | 0 (0) | 0·02 |
| Arms MRC <3/5 | 751 (62) | 0 (0) | 0·04 |
| Independent (mRS pre-stroke) | 1037 (86) | 1 (100) | 0·69 |
| Glucose (median years, IQR) | 7 (5·9 - 8·6) | 9·6 (9·6 - 9·6) | 0·50 |
| SBP (median years, IQR) | 161 (140 - 184) | 119 (119 - 119) | 0·02 |
| Comorbidities |  |  |  |
| Hypertension | 856 (70) | 1 (100) | 0·98 |
| Diabetes | 224 (18) | 0 (0) | 0·07 |
| Dyslipidaemia | 489 (40) | 0 (0) | 0·29 |
| Atrial fibrillation | 429 (35) | 1 (100) | 0·47 |
| Smoking | 105 (9) | 0 (0) | <0·001 |
| Urban hospital | 714 (59) | 0 (0) | 0·02 |
| **12 months post-stroke** | **N=1153**  **n (%)** | **N=63**  **n (%)** |  |
| Age (median years, IQR) | 80 (72 - 87) | 76 (65 - 86) | 0·04 |
| Sex | 540 (47) | 29 (46) | 0·90 |
| Type of stroke |  |  | 0·62 |
| Intracerebral haemorrhage | 138 (12) | 5 (8) |  |
| Cerebral infarction | 959 (83) | 55 (87) |  |
| Stroke unspecified | 54 (5) | 3 (5) |  |
| Stroke severity |  |  |  |
| GCS verbal <5 | 727 (63) | 46 (73) | 0·11 |
| Requires assistance to walk | 494 (43) | 39 (62) | 0·003 |
| Arms MRC <3/5 | 700 (61) | 51 (81) | 0·001 |
| Independent (mRS pre-stroke) | 986 (86) | 52 (83) | 0·42 |
| Glucose (median years, IQR) | 7 (5·9 - 8·7) | 6·9 (5·7 - 8·4) | 0·81 |
| SBP (median years, IQR) | 163 (140 - 185) | 154 (136 - 176) | 0·06 |
| Comorbidities |  |  |  |
| Hypertension | 812 (70) | 45 (71) | 0·87 |
| Diabetes | 206 (18) | 18 (29) | 0·03 |
| Dyslipidaemia | 464 (40) | 25 (40) | 0·93 |
| Atrial fibrillation | 408 (35) | 22 (35) | 0·94 |
| Smoking | 87 (8) | 18 (29) | <0·001 |
| Urban hospital | 679 (59) | 35 (56) | 0·96 |
