# Supplementary material for: The impact of ethnicity on stroke care access and patient outcomes: a New Zealand nationwide observational study
Source: Lancet Reg Health West Pac. 2022 Jan 3;20:100358. doi: 10.1016/j.lanwpc.2021.100358 (PMC8743211; doi:10.1016/j.lanwpc.2021.100358)
Supplement: Supplementary file 5 [file mmc5.docx]

**Supplementary Table 4: Baseline characteristics of patients with and without follow-up data for the non-European cohort**

|  | **Outcomes at follow-up** | **Lost to follow-up** | **p-value** |
| --- | --- | --- | --- |
| **3 months post-stroke** | **N=383**  **n (%)** | **N=173**  **n (%)** |  |
| Age (median years, IQR) | 68 (59 - 79) | 62 (53 - 70) | <0·001 |
| Sex | 197 (51) | 85 (49) | 0·62 |
| Type of stroke |  |  | 0·003 |
| Intracerebral haemorrhage | 57 (15) | 25 (14) |  |
| Cerebral infarction | 302 (79) | 141 (82) |  |
| Stroke unspecified | 24 (6) | 7 (4) |  |
| Stroke severity |  |  |  |
| GCS verbal <5 | 216 (56) | 120 (69) | 0·004 |
| Requires assistance to walk | 153 (40) | 82 (47) | 0·10 |
| Arms MRC <3/5 | 215 (56) | 109 (63) | 0·13 |
| Independent (mRS pre-stroke) | 339 (90) | 161 (96) | 0·03 |
| Glucose (median years, IQR) | 7·2 (6 - 9·6) | 7·3 (6·1 - 10) | 0·81 |
| SBP (median years, IQR) | 156 (136 - 180) | 158 (136 - 177) | 0·90 |
| Comorbidities |  |  |  |
| Hypertension | 276 (72) | 125 (72) | 0·96 |
| Diabetes | 145 (38) | 67 (39) | 0·85 |
| Dyslipidaemia | 177 (46) | 76 (44) | 0·62 |
| Atrial fibrillation | 118 (31) | 51 (29) | 0·75 |
| Smoking | 77 (20) | 51 (29) | 0·02 |
| Urban hospital | 258 (67) | 106 (61) | 0·16 |
| **6 months post-stroke** | **N=272**  **n (%)** | **N=38**  **n (%)** |  |
| Age (median years, IQR) | 70 (60 - 80) | 57.5 (47 - 65) | <0·001 |
| Sex | 142 (52) | 21 (55) | 0·72 |
| Type of stroke |  |  | 0·90 |
| Intracerebral haemorrhage | 43 (16) | 5 (13) |  |
| Cerebral infarction | 210 (77) | 30 (79) |  |
| Stroke unspecified | 19 (7) | 3 (8) |  |
| Stroke severity |  |  |  |
| GCS verbal <5 | 141 (52) | 29 (76) | 0·01 |
| Requires assistance to walk | 105 (39) | 16 (42) | 0·68 |
| Arms MRC <3/5 | 144 (53) | 24 (63) | 0·24 |
| Independent (mRS pre-stroke) | 239 (90) | 37 (97) | 0·11 |
| Glucose (median years, IQR) | 7·2 (6·1 - 9·6) | 6·3 (5·5 - 9·9) | 0·35 |
| SBP (median years, IQR) | 155 (137 - 178) | 148 (130 - 184) | 0·54 |
| Comorbidities |  |  |  |
| Hypertension | 200 (74) | 23 (61) | 0·095 |
| Diabetes | 102 (38) | 16 (42) | 0·58 |
| Dyslipidaemia | 118 (43) | 16 (42) | 0·88 |
| Atrial fibrillation | 87 (32) | 8 (21) | 0·17 |
| Smoking | 45 (17) | 20 (53) | <0·001 |
| Urban hospital | 178 (65) | 22 (58) | 0·36 |
| **12 months post-stroke** | **N=255**  **n (%)** | **N=55**  **n (%)** |  |
| Age (median years, IQR) | 70 (60 - 80) | 59 (51 - 69) | <0·001 |
| Sex | 133 (52) | 30 (55) | 0·75 |
| Type of stroke |  |  | 0·69 |
| Intracerebral haemorrhage | 41 (16) | 7 (13) |  |
| Cerebral infarction | 195 (76) | 45 (82) |  |
| Stroke unspecified | 19 (7) | 3 (5) |  |
| Stroke severity |  |  |  |
| GCS verbal <5 | 130 (51) | 40 (73) | 0·003 |
| Requires assistance to walk | 96 (38) | 25 (45) | 0·28 |
| Arms MRC <3/5 | 134 (53) | 34 (62) | 0·21 |
| Independent (mRS pre-stroke) | 222 (89) | 54 (98) | 0·03 |
| Glucose (median years, IQR) | 7·1 (6·2 - 9·5) | 7·6 (5·2 - 10·3) | 0·49 |
| SBP (median years, IQR) | 157 (137 - 180) | 149 (132 - 179) | 0·27 |
| Comorbidities |  |  |  |
| Hypertension | 186 (73) | 37 (67) | 0·40 |
| Diabetes | 95 (37) | 23 (42) | 0·53 |
| Dyslipidaemia | 111 (44) | 23 (42) | 0·82 |
| Atrial fibrillation | 82 (32) | 13 (24) | 0·21 |
| Smoking | 40 (16) | 25 (45) | <0·001 |
| Urban hospital | 166 (65) | 34 (62) | 0·65 |
